# Application of Radiomics for the Prediction of Radiation-Induced Toxicity in the IMRT Era: Current State-of-the-Art

**DOI:** 10.3389/fonc.2020.01708

**Published:** 2020-10-06

**Authors:** Isacco Desideri, Mauro Loi, Giulio Francolini, Carlotta Becherini, Lorenzo Livi, Pierluigi Bonomo

**Affiliations:** Radiation Oncology, Azienda Ospedaliero-Universitaria Careggi, University of Florence, Florence, Italy

**Keywords:** Radiomics, Intensity modulated radiotherapy, xerostomia, radiation induced lung injury, cardiac toxicity, lower gastro-intestinal toxicity

## Abstract

Normal tissue complication probability (NTCP) models that were formulated in the Quantitative Analyses of Normal Tissue Effects in the Clinic (QUANTEC) are one of the pillars in support of everyday’s clinical radiation oncology. Because of steady therapeutic refinements and the availability of cutting-edge technical solutions, the ceiling of organs-at-risk-sparing has been reached for photon-based intensity modulated radiotherapy (IMRT). The possibility to capture heterogeneity of patients and tissues in the prediction of toxicity is still an unmet need in modern radiation therapy. Potentially, a major step towards a wider therapeutic index could be obtained from refined assessment of radiation-induced morbidity at an individual level. The rising integration of quantitative imaging and machine learning applications into radiation oncology workflow offers an unprecedented opportunity to further explore the biologic interplay underlying the normal tissue response to radiation. Based on these premises, in this review we focused on the current-state-of-the-art on the use of radiomics for the prediction of toxicity in the field of head and neck, lung, breast and prostate radiotherapy.

## Introduction

The seminal QUANTEC collection (1) provided a comprehensive set of recommendations for the estimation of normal tissue complication probability (NTCP) that were largely based on empirical data, whereas the earlier influential paper by Emami (2) was mainly based on a consensus of experts. Leveraging the available published evidence into definitions of dose-volume relationships for most organs at risk epitomized the paradigm shift of QUANTEC.

However, its analyses relied on data from a time when predominantly 3D-conformal radiotherapy (3DCRT) was used with relatively uniform dose distributions. The advent of intensity modulated radiotherapy (IMRT) led to an unprecedented improvement in radiation ballistics (3), allowing for exquisite precision in dose distribution. Over the years, through constant optimization of IMRT techniques (4), the ceiling of organ-at-risk sparing has been reached: in the frame of photon therapy delivery, incremental advances in the reduction of radiation-induced toxicity are unlikely to occur, mainly due to limits dictated by physics. Ideally, further improvement must come from better shaping the dose distribution, which can only be personally optimized if precise knowledge (5) of dose-effect relationships is used. The current state-of-the-art of relying exclusively on NTCP models from QUANTEC has its own caveats (6): above all, the lack of integration of biologic heterogeneity and patients’ individual factors such as age, comorbidities, pre-existing organ dysfunction, and use of systemic agents represent the most limiting factors. In addition, an overarching issue is represented by the paucity of external validation studies (7, 8) for most NTCP models.

Overall, the absence of predictive biomarkers for radiation-related morbidity is a major unmet need in modern radiation therapy. Within the last 10 years (9, 10), the advent of radiomics has reshaped the approach to medical images, based on the hypothesis that they are inherently able to convey information on the underlying physiopathology. Standardization in image acquisition, high-throughput generation of objective descriptors and extensive data-mining characterized the transition from purely qualitative to quantitative imaging (11). As outlined in the pivotal CRUK-EORTC consensus review (12), distinct features can be envisaged in the re-thinking of imaging as a biomarker: non-invasiveness, serial assessment, comprehensive tumor mapping, repeatability, and cost-effectiveness. In the perspective of personalized oncology (13) as currently implemented in the clinic, the use of quantitative imaging may allow us to overcome the known limits associated with molecular profiling. Several applications of radiomics in the field of precision radiation oncology have been identified, providing insights in terms of stage discrimination (14, 15), molecular stratification (16–18), prognostic impact (19, 20), and prediction of response to treatment (21–23). With imaging, the possibility to capture intrinsic tumor and organ-specific heterogeneity could be leveraged to evaluate the individual predisposition to radiation-induced toxicity (24). Thus, radiomics-based analyses have the potential to enrich standard NTCP models for the definition of individualized risk profiling, ultimately aiming for a personalized patient management and optimized therapeutic ratio. At present, such efforts must still be considered investigational and not ready for prime time (25). The aim of our mini-review was to provide an overview on the evidence pertaining to the role of radiomics in the prediction of radiation-induced toxicity for parotid glands, lungs, heart, and rectum. Based on the aforementioned premises, in each of the following sections an introduction on the traditional QUANTEC-based NTCP models is followed by the description of the most relevant data thus far available on radiomics-analyses and their potential in improving the predictive ability of side effects.

## Head and Neck Radiotherapy: Parotid Glands

Xerostomia represents a well known side-effect in head and neck cancer (HNC) radiotherapy (RT), accounting for significant impairment in patients’ quality of life due to its impact on taste, swallowing, and speech (26). The major determinant of xerostomia is radiation-induced damage of the parotid and submandibular glands, which globally release over 80% of saliva (27). The QUANTEC consortium (6, 28) identified a mean parotid gland dose of 26 Gy as a critical threshold for the preservation of salivary function. However, in IMRT clinical practice, it is often challenging to comply with this recommendation, since a detrimental impact on target coverage can’t be completely minimized (29). Furthermore, it has been demonstrated that a late recovery of salivary function is feasible, even in cases of overt xerostomia shortly after RT (30). These considerations led to the assumption that the dose-response relationship of parotid glands is more complex than initially hypothesized in QUANTEC, and that within this context, the use of quantitative imaging could lead to a better understanding of this issue. In an effort to better elucidate radiation-induced xerostomia pathogenesis, van Lujk et al. (31) postulated the existence of stem cell regions in the context of parotid glands involved in the regeneration of salivary function. As the distribution of stem cells within the parotid gland was shown to be inhomogeneous, with the highest concentration located near the dorsal edge of the mandible (where the first branching of the Stensen duct is located), it has been theorized that intentionally sparing these sub-regions would yield better results rather than attempting to spare the whole gland. The validity of this approach was further confirmed by a *post hoc* analysis of the PARSPORT trial performed by Buettner et al. (32). In fact, by taking into account the spatial information of dose distribution within parotid glands, the authors demonstrated that a significantly better prediction of patient-reported xerostomia could be obtained in respect to a model solely based on standard mean dose. Further efforts in unraveling the complex relationship between dose distribution within the parotid and NTCP led to the concept that different thresholds for xerostomia injury and recovery exist. Recently, Guo et al. (33) assessed the spatial radiation dose-based importance pattern in the major salivary glands in relation to late and acute xerostomia in a retrospective population of 146 HNC patients. The authors identified the superior portion of the two parotid glands (low dose region) as the most influential on xerostomia recovery, and demonstrated a different voxel hierarchy pattern for injury and recovery. In a retrospective analysis on 258 patients, Han and colleagues (34) showed an inverse correlation between the pattern of dose-volume histograms and clinical outcomes: a relatively high dose to small portions of a glandular sub-volume (between 10 and 40%) may be more harmful than a low-dose bath effect. Hence, in terms of function preservation, limiting the dose to specific sub-volumes such as the superior-posterior region of the ipsilateral parotid gland may be more useful (nested cross-validation area under the curve (AUC) – values of 0.78 and 0.70 for prediction of injury and recovery, respectively). In this perspective, the identification of quantitative imaging parameters correlated with both acute and late xerostomia is of paramount importance. Changes over time of radiomics features (delta-radiomics) have been extensively evaluated both in terms of acute and late xerostomia prediction (35–38). In an effort to better elucidate the relationship between parotid gland shrinkage after RT and late xerostomia, van Dijk et al. (37) recently demonstrated a correlation between delta radiomics surface changes in contralateral parotid gland and late xerostomia in 68 patients (AUC 0.93 in test cohort). This association was significant during the whole course of RT, but performed best for mid-treatment (week 3). This finding may have profound clinical implications, allowing an early identification of patients at risk for developing late side effects and prompting adaptive re-planning or even switching to other forms of radiation (e.g., proton therapy). A similar approach was performed by Rosen and colleagues (38), who retrospectively analyzed serial cone-beam CTs (CBCT) of 119 HNC cancer patients undergoing RT. The authors concluded that the rate of CBCT-measured parotid gland image feature changes improved NTCP modeling over dose alone for late xerostomia prediction (AUC 0.77). In the context of late xerostomia prediction, baseline evaluation of changes in magnetic resonance (MR) and 18F-fluorodeoxyglucose (FDG) positron emission tomography/computed tomography (PET-CT)-based parotid gland features was also shown to be a promising field of application (39–43). In particular, parotid glands with low metabolic activity and a low fat-to-functional parenchymal ratio were matched by more heterogeneous intensity and texture imaging features: overall, these hypothesis-generating studies showed that pre-treatment radiomics-based prediction outperformed conventional NTCP models. Finally, a machine-learning approach integrating dosiomics, radiomics, and morphological data in predicting both acute and late injury to salivary glands has recently shown promising results (44, 45). Interestingly, by applying a novel artificial intelligence methodology (“likelihood-fuzzy analysis”), Pota et al. (46) identified quantitative predictors of 12-month toxicity through a longitudinal assessment of parotid glands in a dual institution experience. Taking all data together, radiomics-based analyses proved to be reliable tools to assess the risk for xerostomia in HNC patients, warranting further validation in larger prospective cohorts.

## Thoracic Radiotherapy: Lung

Radiation-induced lung injury (RILI) is at the same time a complex radiobiological entity with a multi-faceted physiopathology and a serious challenge for the clinician, representing an important source of morbidity in 15–40% (47, 48) of patients receiving radiation or chemoradiation as definitive treatment for non-small cell lung cancer (NSCLC). In the IMRT era, a stringent trade-off between dose delivery to locoregional disease and adequate sparing of healthy lung tissue is advocated. This assumption was corroborated by a secondary analysis of the controversial RTOG 0617 trial, suggesting that the lack of benefit of dose escalation may have resulted from an increase in cardio-pulmonary mortality in patients receiving more aggressive dose regimens (49, 50). It is well known that RILI is a dose-limiting toxicity in the management of esophageal cancer (51, 52) and lymphoma (53, 54) patients, as well. In view of the usually lower total dose delivered for these malignancies in current practice, the most compelling evidence on radiation-induced lung toxicity can be extrapolated from NSCLC. Hence, it is of primary importance to unravel the intricate network of technical, clinical, and treatment-related factors implicated in the onset of RILI in order to develop models that allow us to accurately predict the risk of serious adverse events. The use of dose estimates to the lung as a predictor of RILI risk is well established (55), while the role of other factors, in particular dose to the heart, is controversial (56–58). Currently, dose-volume parameters, namely the mean lung dose (MLD) and the volume of lungs receiving at least 20 Gy (V20Gy), have been integrated in the QUANTEC (59) as partially reliable surrogates for the risk of radiation pneumonitis. Taking into account the known low dose bath-effect of IMRT, lower dose-volume thresholds have also been suggested, such as V13Gy (60, 61) and V5Gy (54, 62) In comparison with the historical standard Lyman model, the development of the “generalized Lyman-model” (GLM) (63) led to the introduction of a new radiobiological parameter (the effective dose, or D_eff_, corresponding to the equivalent uniform dose, EUD), allowing for exposed volumes of the organ at risk to be weighted differently. However, dose-volume parameters do not ultimately allow us to take into account the functional heterogeneity within different lung regions and among individuals. On the other hand, data extraction from pre-treatment imaging may provide information for a tailored strategy. Thus far, a few reports are available on the potential added value of radiomics in the context of RILI prediction. In a single-center, retrospective experience on 96 patients who received curative RT for esophageal cancer, Anthony et al. (64) evaluated the correlation between the development of symptomatic radiation pneumonitis and pre-treatment analysis of FDG PET/CT and diagnostic CT scans. In a logistic regression model, the addition of the standard uptake value (SUV) standard deviation to 18 lung CT texture feature changes in the low-dose area (0–10 Gy) improved by 0.08 the mean AUC value in discriminating the diagnosis of RILI. In a larger experience on 192 patients treated for NSCLC in the same institution, Krafft et al. (65) extracted 6851 features from planning CT scans, as candidate predictors for RILI. Compared with standard clinical and dosimetric factors, at least absolute shrinkage and selection operator (LASSO) logistic regression, a final 449-feature set of the total lung volume yielded a higher average cross-validated AUC, demonstrating improved discrimination (0.51 and 0.68, respectively). The existence of a strict relationship between the dose distribution, a change of CT texture features before and after RT, and the risk of RILI development was firstly demonstrated by Cunliffe et al. (66). Recently, this dosiomic approach was replicated through a convolutional deep-neural network analysis (67, 68) in a cohort of 70 NSCLC patients treated with volumetric modulated arc therapy (VMAT), providing a high discriminative power (AUC of 0.84) over standard logistic regression models for the prediction of radiation pneumonitis. Taking into account the much less clinically relevant impact of radiation pneumonitis in the context of stereotactic body RT, limited data are available (69, 70) in this context in comparison to conventionally-fractionated regimens. Overall, in parallel to robust prognostic value in the context of stereotactic body RT (71) and chemoradiation (72), the reported data promisingly support the relevance of radiomics in the prediction of lung toxicity. However, to take into account the complexity of RILI, optimal models should integrate, in addition to dosimetric variables, other individual risk factors such as age (73), genetic polymorphysms (74), pre-existing functional impairment of the lung (48), chemotherapy regimens (75), and, curiously, a paradoxical protective effect of smoking as a possible result of functional exhaustion of the inflammatory microenvironment in current smokers (73). In summary, RILI is a multi-faceted phenomenon resulting from complex processes that depend on biologic, dosimetric, and treatment-related variables that need to be integrated in a comprehensive model (76, 77), beyond a mechanicistic dose-response relationship.

## Breast Radiotherapy: Heart

Radiotherapy plays a crucial role in the curative management of non-metastatic breast cancer, with well-established benefits in terms of loco-regional control and survival for node-positive patients (78, 79). In 2005, the Early Breast Cancer Trialists’ Collaborative Group (EBCTCG) meta-analysis on individual patient data epitomized the known potential correlation of radiation and cardiac damage, showing a significant excess of non-breast cancer mortality from heart disease (rate ratio 1.27, SE 0.07, 2p = 0.0001) (80). Notably, the high cure rate of radiation for Hodgkin lymphoma (HL) has been historically offset by late heart dysfunction in long survivors (81). In the QUANTEC publication (82) it was recommended that the heart volume receiving up to 25 Gy (V25) should be below 10%. In current practice, the “ALARA” (“as low as reasonably achievable”) principle is usually applied to left-sided breast cancer patients, aiming for a mean heart dose (MHD) below 2 Gy whenever possible. However, the NTCP model does not take into account other dosimetric factors, such as the possible interaction between cardiac and lung dose-volume parameters (83), as suggested by Cella et al. in an institutional analysis on 90 HL patients (84). Abnormalities in myocardial perfusion and echocardiography have been reported (85) when larger than average heart volumes were inadvertently irradiated. In particular, a mean dose to the left ventricle of 9 + 4 Gy was significantly correlated with a reduced anterior wall strain (-16.8% at 14 months after RT), an early surrogate marker of myocardial function detectable with doppler echocardiographic imaging. Conversely, in patients with relatively low MHD (< 4 Gy), Bian et al. found no association between cardiac dosimetry and left ventricular ejection fraction (LVEF) (86). Multiple heart dose parameters have been associated with clinically relevant cardiotoxicity in breast cancer (87). At a median follow-up of 12 years, Correa et al. found an increased incidence of coronary artery disease and chronic heart failure (CHF) rates for increasing heart dose (85). Likewise, Saiki et al. found a significant association between MHD and the risk of heart failure with preserved ejection fraction (OR: 16.9, 95% CI: 3.9–73.7) (88). In a pivotal study, Darby et al. were able to demonstrate the existence of a linear relationship between the occurrence of major coronary events and MHD, with a 7.4% increase in the risk per Gy (95% CI: 2.9–14.5; *p* < 0.001). Nonetheless, a distinct dose threshold could not be identified (89). In a large cohort of 910 patients, Van den Bogaard et al. confirmed these findings, reporting a 16.5% increase per Gy in the cumulative incidence of acute coronary events (90), although they were not able to detect a correlation between RT dose and LVEF (91). Overall, the inter-individual heterogeneity in cardiac exposure to radiation has been an unresolved issue in cardiotoxicity studies. The inter-observer reproducibility in delineation of heart substructures and their dosimetric evaluation (82) are critical factors for a prospective, personalized risk assessment. Indeed, contouring standardization may have a significant role in minimizing differences in dose reporting (92–95). Patients enrolled in the prospective BACCARAT study (96) underwent a coronary computed tomography angiography (CCTA) before irradiation. By analyzing the dose distribution to the whole heart and its substructures in 89 left-sided subjects, the authors highlighted that MHD is a poor dosimetric surrogate parameter for the left ventricle and coronary arteries (in particular the left anterior descending artery). A machine learning approach based on CCTA-derived radiomics may have potential for a better prediction of atherosclerotic plaques over visual assessment (AUC of 0.73 vs 0.65, *p* = 0.04) (97). Taking all clinical observations together, no NTCP modeling provides conclusive evidence on late heart toxicity based on MHD analysis. To the best of our knowledge, no radiomics applications have been reported for the prediction of radiation-induced heart damage. Interestingly, Currie et al. (98) performed an explorative study based on automated feature extraction from single-photon emission computed tomography (SPECT) imaging in 22 non-cancer patients with cardiomyopathy to evaluate the most potent prognostic index for future cardiac events. With an artificial neural network approach, the authors showed that a ^23^iodine meta-iodobenzylguanidine (^123^I-mIBG) planar global washout higher than 30% was the best indicator for risk of cardiac events when accompanied by a decline in LVEF of more than 10%. In summary, in spite of technical capability of modern IMRT techniques to tightly refine the dose distribution within the thorax, the definition of dose-volume relationships and specific NTCP modeling for myocardial sub-volumes lags behind. Taking into account that the risk of future cardiac events after RT is strongly related to persistent smoking, age, prior cardiac events, and pre-existing cardiovascular risk factors, big data applications (99) may lend support to clinical decision making.

## Prostate Radiotherapy: Rectum

Definitive RT represents one of the main treatment options for localized prostate cancer (100). Thanks to the availability of long-term data on clinical outcome and adverse events, radiation-induced lower gastro-intestinal toxicity remains one of the most relevant factors known to have a detrimental impact on patients’ quality of life (101). The relationship between increased late rectal toxicity and high radiation dose is well known for 3DCRT (102) and conventional fractionation up to 78 Gy, with increasing rates of bleeding with rectal volumes receiving 50, 60, 65, 70, and 75 Gy greater than 50, 35, 25, 20, and 15%, respectively (V50Gy > 50%, V60Gy > 35%, V65Gy > 25%, V70Gy > 20%, and V75Gy > 15%) (103, 104). When externally validated in patients treated with 3DCRT, the QUANTEC-based EUD model had relatively low predictive power (AUC 0.61) for late rectal bleeding (105). Further, the NTCP cross-applicability to IMRT for chronic gastrointestinal toxicity was assessed in a large single-institution cohort study (106). Indeed, debilitating symptoms such as fecal incontinence or rectal urgency were mostly reported when large volumes of the rectum were exposed to intermediate doses, as confirmed by the Medical Research Council RT01 randomized phase 3 trial (107) and the long-term follow-up of the AIROPROS 0102 study (108). In recent years, the implementation of moderate hypo-fractionated regimens in clinical practice prompted the development of dose-volume constraints adapted to different treatment schedules (109). Unlike what happens for moderate hypo-fractionated IMRT, high rather than low-dose regions in the rectum predict toxicity after an ultra hypo-fractionated regimen. Of note, V35Gy was shown to be a strong predictor of rectal bleeding (110) and a recent pooled analysis of patients treated within four different trials demonstrated that late toxicity and quality of life were significantly related to V38Gy after the delivery of 35-40 Gy in five fractions (111). Overall, prospectively defined dosimetric predictors of lower gastro-intestinal toxicity can be adapted according to different techniques and fractionations used in the context of definitive treatment for localized prostate cancer.

In view of the available spectrum of NTCP models and of the clinical variability of late rectal side effects, extracting mineable data from imaging would facilitate a personalized treatment prescription. Few radiomics analyses allow us to refine the toxicity prediction in the current scenario. In a single-center prospective study on 33 patients treated with moderately accelerated IMRT (70.2 Gy in 26 fractions), Abdollahi et al. (112) performed a machine learning approach on pre- and post-treatment T2-weighted MR scans of the rectal wall. Out of a total of 1096 features, a 37-set of descriptors extracted from baseline T2-weighted images was more accurate (mean AUC of 0.68) than post-treatment T2-weighted apparent diffusion coefficient (ADC) and delta values. Of note, a broad clinical endpoint was chosen by the authors (G1 rectal toxicity, occurring in 54% of the cohort). Similar pilot analyses from the same group focused on the bladder wall (113) and femoral head changes (114). In a secondary analysis of the multi-institutional randomized HYPRO trial, Rossi et al. (115) evaluated the correlation of late gastrointestinal and genitourinary toxicity with non-treatment related characteristics (age, baseline PSA, Gleason score, comorbidities), DVH parameters, and radiomics features. Of the 820 patients with intermediate and high risk prostate cancer enrolled in the trial, 351 had dose distributions to rectum and bladder available for 3D texture analysis. For both rectal bleeding and fecal incontinence, logistic NTCP models showed that the addition of texture features led to a statistically significant improvement in the predictive ability (AUC of 0.73 for both; *p* < 0.04), higher than what was obtained with clinical and DVH parameters. In a smaller prospective study on 64 patients, Mostafaei et al. came to similar results by analyzing baseline CT markers with a stacking regression algorithm (116). Interestingly, an explorative approach focused on four patients irradiated on a 1.5 Tesla MR-Linear Accelerator within a prospective observational trial. Delta-radiomics assessed with a longitudinal T2-weighted intensity histogram of prostate and surrounding organs at risk showed early significant variations of the rectal wall, with change in mean, median, and standard deviation metrics values at the second week of treatment. A longitudinal radiomic data acquisition process was deemed feasible on the hybrid machine (117). To summarize, in the modern context of prostate RT, the prediction of gastrointestinal toxicity based only on NTCP models may be misleading, given the current trend for dose-escalated IMRT and the establishment of hypo-fractionated and ultra hypo-fractionated regimens as standards of care. Early prospective data on the integration of radiomics analyses are available. Potentially, these features may represent a valuable tool for clinical decision in the future. Further refinement could be provided by applying machine learning methods and bioinformatics tools to genome-wide data to identify patients with a greater congenital risk of toxicity before treatment (118).

## Assessing the Quality of Radiomics Investigations: A Word of Caution

In the previous sections, the potential of radiomics for the prediction of radiation-induced toxicity for parotid glands, lung, heart, and rectum was highlighted. Promisingly, quantitative imaging represents an area of active research under the light of precision oncology (25). Nonetheless, when evaluating the investigations thus far published on radiomics, some caveats need to be taken into account. In view of the complexity of the radiomic workflow, Lambin and colleagues (11) introduced a radiomics quality score (RQS) tool. Based on a set of 16 well-defined criteria addressing several aspects such as image protocol quality, segmentation method, feature reduction, presence of biologic correlates, and extent of validation, the authors proposed to define an objective ranking of quality for radiomics studies. In particular, a score of 36 corresponds to the highest value achievable, whereby the prospective validation of a radiomics signature in a registered trial confers the largest contribution (7 points). Through a systematic review of the literature focusing on the link between radiomic biomarkers and tumor biology, Sanduleanu et al. (119) applied the RQS in 41 studies. Unsurprisingly, most studies (30/41) were of poor quality, with an average score of 30% or less, mainly because of a lack of robust segmentation, external validation, and discrimination based on cut-off values. In addition, interobserver variability among authors in terms of scoring was significant, suggesting that the proposed scale requires further refinement. When applying the RQS to evaluate the methodological quality of the most relevant radiomics analyses thus far published for the prediction of xerostomia, RILI, and late rectal toxicity, the overall outlook (Tables 1, 2) is unsatisfactory. Although all studies performed well in terms of describing feature reduction methods (all used measures to decrease the risk of overfitting), multivariable analyses with non-radiomic factors, and reporting cut-off analyses, the weaknesses are represented by the limited validation (typically, on a dataset from the same institution), the retrospective study design, the infrequent discussion of biological correlates, and the lack of cost-effectiveness. A notable exception is represented by the work of Rossi et al. (112) with a RQS of 80.5% (29/36): the high score can be justified due to the fact that the radiomics signature in this study was prospectively validated in a large, multi-institutional randomized trial with a resulting potential direct clinical utility. In view of the suboptimal methodological quality frequently observed in the radiomics studies we evaluated, caution is advised in the interpretation of the reported findings. Another relevant limit to bear in mind is the lack of standardization in regards to imaging features definition and interpretation. In this perspective, the recently published Image Biomarker Standardization Initiative (IBSI) position paper (120) should be viewed as a relevant step ahead, fostering homogeneity in radiomics analyses across different research platforms.

**TABLE 1:** Most consolidated data in radiomics-based approaches in predicting radiotherapy-induced xerostomia.

Reference	Patient population	Study design	Imaging biomarker	Main outcome	Main finding	Prediction measure	RQS
Van Dijk (37)	68	Retrospective (training and test cohort)	CT	Late Xerostomia	Contralateral PG surface change @ 3 wks	AUCtrain = 0.92; AUCtest = 0.93	50%
Rosen (38)	119	Retrospective (single cohort)	CBCT	Late Xerostomia	Contralateral PG shrinkage	AUC = 0.77	38.8%
Guo (33)	146	Retrospective (Single cohort)	CT	Late xerostomia	Low dose region to superior portion of the 2 PGs	AUC = 0.68	36.1%
Han (34)	589 (258 assessable for late xerostomia)	Prospective (multicentric)	CT	Late Xerostomia	Low dose bath to whole gland	AUC = 0.70	50%
Van Dijk (41)	68 (25 patients for external cohort)	Retrospective (+ validation cohort)	MR	Late Xerostomia	High intensity MR P90 values	AUC = 0.83	50%
Wilkie (42)	47	Prospective (single cohort)	PET	Late Xerostomia	Pre-treatment PET P90 values	AUC = 0.78	44.4%

*CT, computed tomography; PG, parotid gland, AUC: area under the curve; CBCT, cone-beam CT; MR, magnetic resonance; P90, 90th percentile; PET, positron-emission tomography; RQS, radiomics quality score.*

**TABLE 2:** Most promising data in radiomics-based approaches in predicting radiotherapy-induced toxicity in the treatment of solid tumors.

Reference	Patient population	Study design	Imaging biomarker	Main outcome	Main finding	Prediction measure	RQS
Krafft (65)	192 (NSCLC)	Retrospective (single cohort)	CT	RILI	449-feature set of the total lung volume	AUC = 0.68	44.4%
Liang (67)	70 (NSCLC)	Retrospective (single cohort)	CT	RILI	GLCM of ipsilateral lung	AUC = 0.78	41.6%
Rossi (115)	351 (PCA)	Prospective	CT	Late Rectal toxicity	42 Texture features (LRHGE most selected	AUC = 0.73	80.5%
Abdollahi (112)	33 (PCA)	Prospective	MR	Late Rectal toxicity	Pre-treatment T2 MRI features	AUC = 0.68	25%

*NSCLC, non-small cell lung cancer; CT, computed tomography; RILI, radiation-induced lung injury; AUC, area under the curve; GLCM, gray level co-occurrence matrix; PCA, prostate cancer; LRHGE, long run high Gaey level emphasis; MR, magnetic resonance; RQS, radiomics quality score.*

## Conclusion

In comparison to efficacy outcomes, the current state-of-the-art on radiomics prediction of radiation-induced toxicity is still relatively limited, with the notable exception of xerostomia prognostication (Tables 1, 2). Taking all data together, the vast majority of reviewed studies suggested that indeed radiomics applications may increase the predictive ability of organ-specific side effects over standard clinical and dosimetric factors. For further progress, four major areas of improvement can be envisaged. Firstly, the need for standardization is a critical, well-recognized major step for further development (120, 121). Secondly, in view of the frequent single-center retrospective design and the generally low number of enrolled patients and of clinical endpoints (i.e., side effects), the robustness of data is questionable for most studies (122). In this perspective, the lack of or very limited external validation in independent datasets is a point of weakness for both conventional NTCP models (123) and radiomics applications (119). Thirdly, progress in the field of radio-genomics is eagerly awaited (124), in order to improve the understanding of underlying biological processes, such as intrinsic radio-sensitivity. Lastly, controlled randomized clinical trials testing radiomics-based interventions in adequately powered studies are still yet to be published. At present, no single radiomics finding is readily applicable to patient management in clinical practice. Nonetheless, the available body of evidence is encouraging and warrants further investigation, given the size of benefit demonstrated in terms of high predictive ability of common toxicities. In conclusion, building on established NTCP models, the so far available hypothesis-generating data underline the potential of radiomics for improved clinical decision making in precision radiation oncology.

## Author Contributions

All authors listed have made a substantial, direct and intellectual contribution to the work, and approved it for publication.

## Conflict of Interest

The authors declare that the research was conducted in the absence of any commercial or financial relationships that could be construed as a potential conflict of interest.
